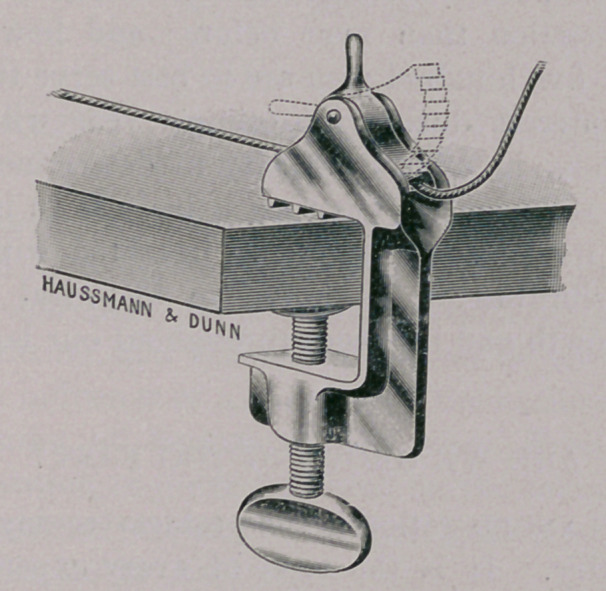# Department of Canine and Feline Medicine and Surgery

**Published:** 1900-10

**Authors:** Cecil French

**Affiliations:** Washington, D. C.


### DEPARTMENT OF CANINE AND FELINE MEDICINE AND SURGERY.

By Cecil French, D.V.S.,
WASHINGTON, D. C.

### THE DAWSON-FRENCH CANINE HOPPLES.

These hopples are constructed on a plan adapted to canine and feline purposes, and were suggested to me by a contrivance devised by Dr. Charles Dawson, formerly attached to the Department of Pathology, Bureau of Animal Industry, for the purpose of controlling the smaller animals used for inoculation experiments. Messrs. Haussman & Dunn have very kindly interested themselves in my endeavor to secure the production of a perfected article. The main feature of the hopples is their self-locking action. A swinging ratchet arrangement is suspended in a frame through which the control rope passes. The frame is supplied with a screw-clamp by means of which it is attachable to and removable from any table at will. One set of four large and one set of four small noose leg-bands are provided. These will fit any sized animal, and are connected to the control ropes by steel snaps. Should the operator wish to tighten the control rope he does so by merely pulling on it, and the moment he lets it go it is firmly clinched by the ratchet. The animal can be quickly released from the control position at any moment by simply holding back the handle bars, by which the ratchet is prevented from clinching and allows free passage for the rope.

## Figures and Tables

**Figure f1:**